# Effect of cholecalciferol on serum hepcidin and parameters of anaemia and CKD-MBD among haemodialysis patients: a randomized clinical trial

**DOI:** 10.1038/s41598-020-72385-w

**Published:** 2020-09-23

**Authors:** Yoshitsugu Obi, Satoshi Yamaguchi, Takayuki Hamano, Yusuke Sakaguchi, Akihiro Shimomura, Tomoko Namba-Hamano, Satoshi Mikami, Osamu Nishi, Motoko Tanaka, Akihito Kamoto, Yasue Obi, Naohisa Tomosugi, Yoshiharu Tsubakihara, Yoshitaka Isaka

**Affiliations:** 1 Department of Nephrology, Osaka University Graduate School of Medicine, Suita, Osaka Japan; 2 Obi Clinic, Osaka, Osaka Japan; 3 Division of Nephrology, University of Tennessee Health Science Centre, Memphis, TN USA; 4 Department of Inter-Organ Communication Research in Kidney Disease, Osaka University Graduate School of Medicine, 2-2 Yamada-oka, Suita, Osaka 565-0871 Japan; 5 Department of Nephrology, Nagoya City University Graduate School of Medical Science, Nagoya, Japan; 6 Department of Internal Medicine, Higashikouri Hospital, Hirakata, Osaka Japan; 7 Nishi Clinic, Osaka, Osaka Japan; 8 Akebono Clinic, Kumamoto, Kumamoto Japan; 9 Futaba Clinic, Osaka, Osaka Japan; 10 Division of Advanced Medicine, Medical Research Institute, Kanazawa Medical University, Kawakita, Ishikawa Japan; 11 Medical Care Proteomics Biotechnology Co., Ltd, Kawakita, Ishikawa Japan; 12 Division of Management in Health Care Sciences, Graduate School of Health Care Sciences, Jikei Institute, Osaka, Osaka Japan

**Keywords:** Renal replacement therapy, Metabolic syndrome

## Abstract

In this multicentre double-blind randomized clinical trial, we investigated the effects of oral cholecalciferol supplementation on serum hepcidin and parameters related to anaemia and CKD-MBD among haemodialysis patients. Participants were assigned in a 2:2:1:1 ratio to either (1) thrice-weekly 3,000-IU cholecalciferol, (2) once-monthly cholecalciferol (equivalent to 9,000 IU/week), (3) thrice-weekly placebo, or (4) once-monthly placebo. We also examined the effect modifications by selected single nucleotide polymorphisms in vitamin D-related genes. Out of 96 participants, 94 were available at Month 3, and 88 completed the 6-month study. After adjustment for baseline values, serum hepcidin levels were higher at Day 3 in the combined cholecalciferol (vs. placebo) group, but were lower at Month 6 with increased erythropoietin resistance. Cholecalciferol increased serum 1,25(OH)_2_D levels, resulting in a greater proportion of patients who reduced the dose of active vitamin D at Month 6 (31% vs. 10% in the placebo group). Cholecalciferol also suppressed intact PTH only among patients with severe vitamin D deficiency. In conclusion, cholecalciferol supplementation increases serum hepcidin-25 levels in the short term and may increase erythropoietin resistance in the long term among haemodialysis patients. Both thrice-weekly and once-monthly supplementation effectively increases serum 1,25(OH)_2_D levels, and hence, reduces active vitamin D drugs.

**Clinical Trial Registry**: This study was registered at ClinicalTrials.gov and University Hospital Medical Information Network Clinical Trials Registry (UMIN-CTR) as NCT02214563 (registration date: 12/08/2014) and UMIN000011786 (registration date: 15/08/2014), respectively (please refer to the links below). ClinicalTrials.gov: https://clinicaltrials.gov/ct2/show/record/NCT02214563. UMIN-CTR: https://upload.umin.ac.jp/cgi-open-bin/ctr/ctr.cgi?function=brows&action=brows&type=summary&recptno=R000017152&language=E.

## Introduction

Vitamin D plays a pivotal role in maintaining bone^1,2^ and mineral homeostasis^3,4^. Vitamin D levels decrease with poor dietary intake and low UV-B exposure^5^, and inflammatory status (i.e., high interleukin-6 [IL-6]) also inhibits the synthesis of vitamin D-binding protein (DBP) in the liver. These factors are frequently observed among patients with advanced chronic kidney disease (CKD)^6,7^, and hence, vitamin D deficiency is highly prevalent in this population^8–10^. Vitamin D deficiency in CKD accelerates the development of mineral and bone disorder (MBD) and has been associated with adverse clinical outcomes such as anaemia^11,12^, cardiovascular events^13^, and mortality^13,14^.

Although the link between vitamin D deficiency and anaemia can be attributed to those precipitating factors (e.g., poor nutritional status, chronic illness, and inflammation), previous clinical studies suggested that vitamin D supplementation may improve the response to erythropoiesis-stimulating agents (ESAs)^15^. Postulated mechanisms include the immunomodulatory property and the hepcidin-lowering effect of vitamin D. Indeed, a short-term study of healthy subjects has shown that a single high-dose cholecalciferol supplementation markedly suppressed serum level of hepcidin^16^, a type II acute phase protein that decreases iron availability via iron sequestration^17^. In addition, activation of the vitamin D receptor transcription factor was reported to stimulate the growth of erythroid progenitors^18^, and the resultant iron consumption can also lower hepcidin levels.

Previous observational studies have suggested pleiotropic effects of vitamin D among pre-dialysis chronic kidney disease (CKD) patients^19,20^, kidney transplant recipients^21,22^, and dialysis patients^23^, and the current guidelines suggest vitamin D supplementation for pre-dialysis CKD patients with vitamin D deficiency^24^. However, clinical trials have not shown any clear benefits on clinical outcomes so far. Additionally, previous studies showed mixed data about the anti-inflammatory effect of vitamin D, and there are scarce data on its efficacy for the management of anaemia among patients with end-stage kidney disease (ESKD) despite their ability to convert 25-hydroxyvitamin D [25(OH)D] to 1,25-dihydroxyvitamin D [1,25(OH)_2_D] via monocyte 1α-hydroxylase^25^. It is also important to develop an administration protocol that minimizes the pill burden and to identify which patients are more likely to benefit from vitamin D supplementation.

Therefore, we performed a multicentre double-blind randomized control trial (RCT) to evaluate the efficacy of cholecalciferol supplementation on serum hepcidin levels and CKD-MBD parameters among ESKD patients on maintenance haemodialysis (MHD). This study was feasible particularly in Japan where most patients were not evaluated for their vitamin D status or supplemented with cholecalciferol because serum 25(OH)D measurement and cholecalciferol are reimbursed only for vitamin D deficiency-induced osteomalacia/rickets and the prevention of denosumab-induced hypocalcaemia, respectively. We also examined whether the effects of cholecalciferol were modified by administration interval and selected single nucleotide polymorphisms (SNPs) in vitamin D-related genes.

## Methods

### Study design & participants

This is a multicentre double-blind randomized clinical trial. Participants were recruited from thrice-weekly MHD patients receiving erythropoiesis-stimulating agents at eight dialysis facilities in Japan between August 1st, 2014 and September 30th, 2015. Exclusion criteria included the use of native vitamin D supplements (i.e., cholecalciferol or ergocalciferol), hypercalcemia (i.e., corrected calcium ≥ 10.5 mg/dL), and intravenous iron use. Patients with receiving continuous erythropoiesis receptor activator (i.e., epoetin β pegol), which suppresses serum hepcidin levels for a much longer time than the other ESAs, were excluded. This study was approved by the ethics committees of Osaka university and Higashikouri hospital and was performed in accordance of Declaration of Helsinki. All participants gave written informed consent. This study was registered at ClinicalTrials.gov and University Hospital Medical Information Network Clinical Trials Registry (UMIN-CTR) as NCT02214563 (registration date: 12/08/2014) and UMIN000011786 (registration date: 15/08/2014), respectively.

### Intervention

Patients were randomly assigned in a 2:2:1:1 ratio to either (i) thrice-weekly 3,000-IU cholecalciferol, (ii) once-monthly cholecalciferol (equivalent to 9,000 IU/week; either 36,000 IU or 45,000 IU as appropriate), (iii) thrice-weekly placebo, or (iv) once-monthly placebo. The experimental cholecalciferol and placebo capsules were provided by Molecular Physiological Chemistry Laboratory, Inc. (Tokyo, Japan); cholecalciferol was dissolved with olive oil and coated by soft capsule made of gelatine and glycerine whereas placebo contained olive oil without cholecalciferol. Stratified randomization according to serum albumin levels (< or ≥ 4.0 mg/dL) was performed with the use of a computer-generated random-sequence. All patients started the study at the first dialysis session of the week (i.e., either Monday or Tuesday). During the 6-month study period, cholecalciferol or placebo was given after dialysis session thrice weekly or once monthly as per the assignment. Adherence was ensured via direct observation by medical staff. Both participants and physicians were unaware of the individual treatment assignments. Other than the study intervention, the use of intravenous iron and nutritional vitamin D, either prescription or over-the-counter products, was prohibited throughout the trial. Physicians were eligible to change doses of cinacalcet, active vitamin D drugs, oral iron, and ESAs if indicated by the clinical practice guidelines.

As ESA treatment, either epoetin-α, epoetin-β, or darbepoetin-α was intravenously administered at the end of haemodialysis session. Typically, epoetin-α and epoetin-β were given at each session whereas darbepoetin-α was given at every Monday/Tuesday in six dialysis units and every Wednesday/Thursday in the remaining two dialysis units.

### Outcomes

The primary outcomes were serum hepcidin-25 levels at Day 3 and Month 3. Day 3 was selected based on a previous study showing ergocalciferol treatment suppressed serum hepcidin levels at least up to 72 h^16^. We used Month 3 to evaluate its long-term effectiveness. We also evaluated serum hepcidin-25 levels at Month 6 as the secondary outcome. Other secondary outcomes include (i) percent changes of ESA resistance index; (ii) pre-dialysis blood concentrations of inflammatory markers (i.e., high-sensitive CRP, interleukin-6 [IL-6], and tumor necrosis factor α [TNF-α]); (iii) pre-dialysis blood concentrations of CKD-MBD parameters (i.e., calcium, phosphate, intact parathyroid hormone, 1,25(OH)_2_D, bone specific alkaline phosphatase [BSAP], and tartrate-resistant acid phosphatase 5b). ESA resistance index was calculated as weekly darbepoetin dose (μg)/ haemoglobin (g/dL)/ post-dialysis weight (kg), after converting epoetin-α/β dose to the equivalent darbepoetin-α dose (200 IU epoetin-α/β = 1 μg darbepoetin-α). At Day 3, we measured only serum hepcidin-25, IL-6, and TNF-α levels.

### Measurements

All blood samples were drawn before the first dialysis session of the week and sent for laboratory tests except for serum 25(OH)D and hepcidin-25. Those samples were then stored at − 80 °C for serum 25(OH)D and hepcidin-25 measurement after the end of the trial. Serum 25(OH)D and hepcidin-25 levels were measured using DiaSorin Liaison chemiluminescence immunoassay and liquid chromatography coupled with tandem mass spectrometry, respectively.

### Genetic variants

In order to evaluate the impact of gene variants on the effects of cholecalciferol, we performed genome-wide SNP genotyping used Affymetrix Japonica genotyping array v1.0 (Toshiba Corp., Japan), which contains 659,636 SNPs based on the linkage-disequilibrium structure of 1,070 Japanese individuals with whole genome sequencing data. Among 96 study participants, 89 (93%) provided separate written informed consents for the evaluation of their genetic variants. Heparinized whole blood was sent to the analytics centre. Among those vitamin D-related genes that were reported to be associated with serum 25(OH)D levels, we selected the following genes with minor allele frequency > 5%: DBP (rs7041, rs12512631, and rs2282679), CYP2R1 (rs2060793), CYP24A1 (rs2209314), and VDR (rs11568820). All of these SNPs were directly determined without imputation.

### Statistical analyses

We analysed outcomes in accordance with the intention-to-treat principle. We combined the thrice-weekly and once-weekly regimens, and then compared the cholecalciferol vs. placebo group in the primary analyses. Given the risk of bias due to the relatively small number of patients that may induce between-group imbalance at baseline, the effects of cholecalciferol were determined with adjustment for baseline values of the outcome variable in multivariate linear regression models as per the study protocol. We also examined the difference in the treatment effect on serum hepcidin-25 levels between thrice-weekly vs. once-monthly administration by comparing 3 categories of intervention (i.e., thrice-weekly cholecalciferol, once-monthly cholecalciferol, and combined placebo) in the multivariable model.

We conducted non-prespecified post-hoc analyses as follows. First, we conducted stratified analyses for serum hepcidin-25 levels at Day 3 and Month 3 according to clinical characteristics. We then examined the effect modification by including an interaction term with the intervention variable into the multivariate model. Second, changes in the dose of active vitamin D drugs from baseline to Month 6 was compared between the cholecalciferol and placebo groups. We used 3 categories for the dose change; decrease, unchanged, or increase. Decrease in active vitamin D drugs was defined as a dose reduction or a route change from oral to intravenous whereas increase in active vitamin D drugs was defined in an opposite manner. One patient changed the route from oral to intravenous and another patient changed from intravenous to oral during the study period. Between-group difference was evaluated by the Wilcoxon–Mann–Whitney rank sum test. Third, we evaluated how the effect of cholecalciferol on intact PTH changed according to baseline serum 25(OH)D levels using the multivariable fractional polynomials interaction method. Since intact PTH can be largely affected by cinacalcet treatment, we restricted this post-hoc analysis to those patients who received 12.5 mg/day or less cinacalcet at baseline. Fourth, the association between baseline serum 25(OH)D levels and the number of each minor allele (i.e., 0, 1, or 2) was evaluated in the multivariate linear regression model with adjustment for age, sex and season of blood draw in the analysis. Lastly, we examined the effect modification by vitamin D-related gene variants using interaction terms for serum hepcidin-25 levels at Day 3 and Month 3 as well as serum 1,25(OH)_2_D levels at Month 3. Statistical significance in the associations of those gene variants were evaluated by the trend test.

Estimated effects of cholecalciferol were presented as absolute values for normally distributed outcome variables whereas we used relative ratios after exponentiating coefficients for right-skewed variables. The only exception was serum 25(OH)D; it showed a right-skewed distribution but the effect of cholecalciferol was presented as absolute values based on previous studies. All statistical analyses were conducted using Stata IC 14 (Stata Corp., TX, USA).

### Sample size calculation

A previous study reported that serum hepcidin levels were 50.1 ± 23.9 ng/mL among ESKD patients on MHD^26^. Since cholecalciferol supplementation was reported to reduce serum hepcidin levels by 32% among healthy subjects^16^, we hypothesized that serum hepcidin levels at Month 3 would be 50.1 ± 23.9 ng/mL and 34.1 ± 16.3 ng/mL in the placebo and cholecalciferol arm, respectively. Assuming a dropout rate of 25%, a sample size of 90 patients (60 in cholecalciferol group and 30 in placebo group) was provided to obtain > 80% power with an alpha level of 0.05.

### Previous presentation

Preliminary results of this study have been partly presented as posters at the Kidney Week 2016 of American Society of Nephrology, Chicago, United States.

## Results

### Baseline characteristics

**Figure 1:**
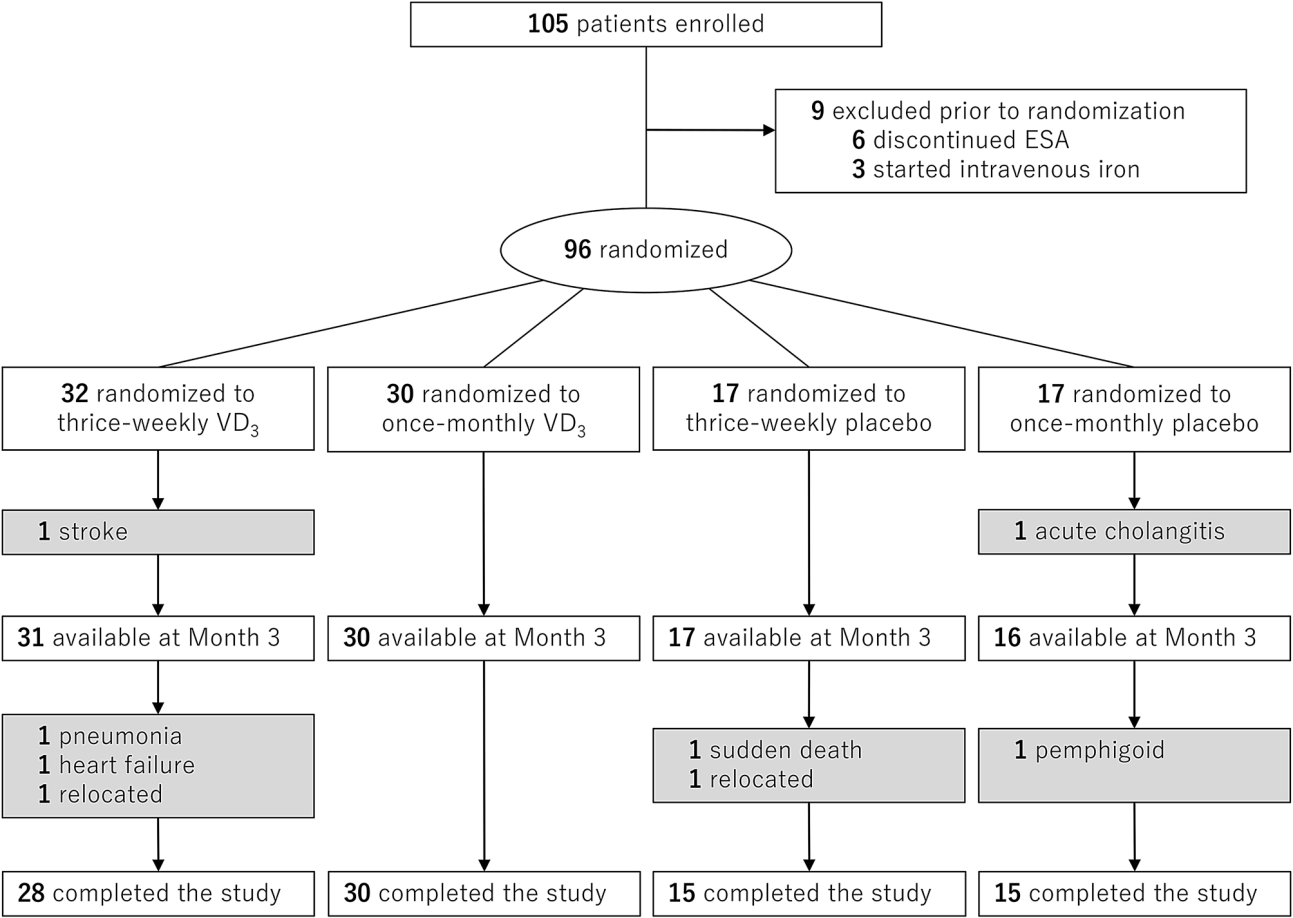
Study flow diagram.

We enrolled 105 patients at 8 facilities; 9 were withdrawn prior to randomization and the remaining 96 were randomly assigned to thrice-weekly cholecalciferol (n = 32), once-monthly cholecalciferol (n = 30), thrice-weekly placebo (n = 17) or once-monthly placebo (n = 17) (Fig. 1). Overall, the median (interquartile range, IQR) of age was 67 (59, 77) years, among whom 64% were male. The median (IQR) of dialysis vintage was 4.9 (2.5, 8.1) years. Baseline serum hepcidin-25 and 25(OH)D showed right-skewed distributions and were median 21.6 (5.4, 43.6) ng/mL and 10.9 (8.3, 13.9) ng/mL, respectively. The combined cholecalciferol vs. placebo groups were well-balanced at baseline except for serum 25(OH)D levels and the prevalence of calcium carbonate use, both of which were lower in the cholecalciferol group (*P* = 0.01 and 0.05, respectively) (Table 1). Supplementary Table S1 online shows baseline characteristics across 3 groups (i.e., thrice-weekly cholecalciferol, once-monthly cholecalciferol, and placebo).

**Table 1:** Baseline characteristics of study participants.

	Placebo (n = 34)	Cholecalciferol (n = 62)
Age (years)	67 (55, 73)	67 (61, 78)
Male (%)	65%	63%
Dialysis vintage (years)	5.8 (3.7, 12.1)	4.0 (2.5, 7.9)
Post-dialysis weight (kg)	58.3 (51.0, 69.5)	57.5 (49.2, 67.5)
**Comorbidities**		
Hypertension (%)	85%	76%
Hyperlipidaemia (%)	44%	35%
Diabetes (%)	47%	52%
Cardiovascular disease (%)	35%	35%
Liver disease (%)	6%	6%
**Laboratory measurements**		
Haemoglobin (g/dL)	10.4 ± 1.0	10.8 ± 0.9
Albumin (g/dL)	3.7 ± 0.3	3.7 ± 0.3
Creatinine (mg/dL)	11.0 (8.7, 11.6)	10.5 (9.2, 12.5)
Calcium (mg/dL)	9.0 ± 0.6	9.0 ± 0.6
Phosphate (mg/dL)	4.9 (4.1, 5.7)	5.0 (4.4, 5.7)
Intact PTH (ng/mL)	93 (51, 183)	120 (65, 190)
BSAP (μg/L)	25.9 (21.7, 33.7)	28.6 (23.2, 35.7)
TRACP-5b (mU/dL)	386 (263, 610)	437 (294, 672)
25-hydroxyvitamin D (ng/mL)	12.1 (9.6, 15.1)	9.9 (8.0, 12.4)
1,25-dihydroxyvitamin D (pg/mL)	14 (9, 21)	15 (10, 20)
Transferrin saturation (%)	24 (16, 31)	22 (18, 29)
Ferritin (ng/mL)	47 (21, 101)	62 (36, 95)
**Medication**		
Oral iron treatment (%)	3%	3%
**ESA type (%)**		
Epoetin-α/β	21%	32%
Darbepoetin-α	79%	68%
ACE inhibitors or ARB (%)	53%	53%
Calcium carbonate (%)	65%	44%
NCC phosphate binders (%)	76%	76%
Active vitamin D drugs (%)	85%	87%
Cinacalcet (%)	26%	31%

Data are presented as %, mean ± SD, or median (IQR) as appropriate.

*ACE* angiotensin converting enzyme, *ARB* angiotensin II receptor blockers, *BSAP* bone-specific alkaline phosphatase, *ESA* erythropoiesis-stimulating agent, *NCC* non-calcium containing, *PTH* parathyroid hormone, *TRACP-5b* tartrate-resistant acid phosphatase 5b.

ESA resistance index was calculated as weekly darbepoetin dose (μg)/ hemoglobin (g/dL)/ dry weight (kg) after converting epoetin-α/β dose to the equivalent darbepoetin-α dose (200 IU epoetin-α/β = 1 μg darbepoetin-α).

### Safety

Out of 96 participants, 4 patients in the placebo group and 5 patients in the cholecalciferol group dropped out and became unavailable for analyses due to adverse events; 1 sudden death, 1 acute cholangitis, and 1 bullous pemphigoid in the placebo group, and 1 congestive heart failure and 1 traumatic subarachnoid haemorrhage in the cholecalciferol group (Fig. 1). The other adverse events were 1 infection episode and 1 small bowel obstruction in the placebo group and 2 infection episodes, 1 atherosclerosis obliterans, 1 spinal compression fracture, and 1 bladder cancer in the cholecalciferol group. No patients developed hypercalcemia (≥ 10.5 mg/dL) during the follow-up period.

### Effect of cholecalciferol on serum 25(OH)D

**Figure 2:**
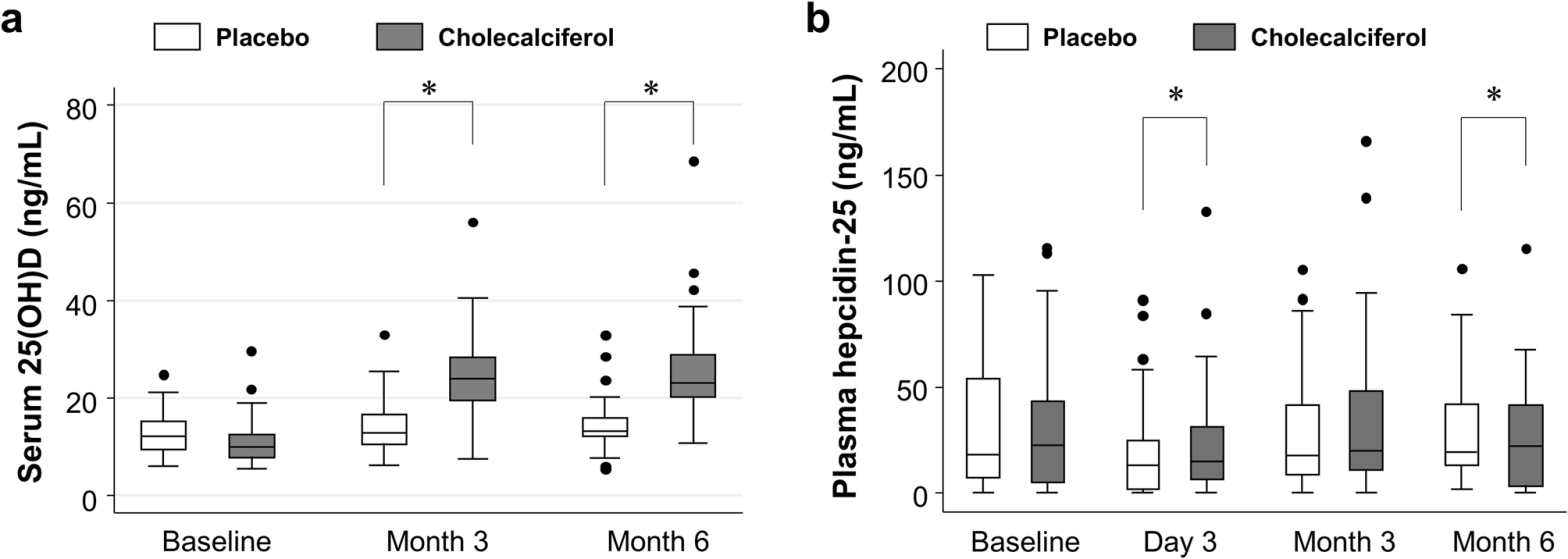
Changes in (**a**) serum 25-hydroxyvitamin D and (**b**) hepcidin-25 levels in the placebo and cholecalciferol groups. *P < 0.05 with adjustment for baseline values.

Median (IQR) serum 25(OH)D levels at Day 3 were 13.5 (9.0, 15.4) ng/mL, 12.1 (9.7, 14.6) ng/mL, and 15.2 (12.7, 18.8) ng/mL in the placebo, thrice-weekly cholecalciferol, and once-monthly cholecalciferol group, respectively. At Month 3 and 6, there was no significant difference between thrice-weekly vs. once-monthly cholecalciferol groups (*P* = 0.19 and 0.40, respectively), and both groups showed higher serum 25(OH)D levels than the placebo group (*P* < 0.001). Median (IQR) serum 25(OH)D levels at Month 3 and 6 were 12.9 (10.6, 16.6) ng/mL and 13.2 (12.2, 16.0) ng/mL in the placebo group, respectively, and were 23.9 (19.5, 28.3) ng/mL and 23.2 (20.4, 28.9) ng/mL in the combined cholecalciferol group, respectively (Fig. 2a).

### Effects of cholecalciferol on serum hepcidin, ESA resistance, and inflammatory markers

Among 69 darbepoetin users at baseline, 38 and 31 patients were given darbepoetin at the first and second dialysis session of the week (i.e., Monday/Tuesday and Wednesday/Thursday), respectively. During the follow-up, 2 and 3 patients in the placebo group converted ESA from darbepoetin to epoetin-α between Day 3 and Month 3 and between Month 3 and Month 6, respectively, and so did 2 and 2 patients in the cholecalciferol group. One patient in the placebo group converted ESA from epoetin-α to darbepoetin between Day 3 and Month 3. Table 2 shows median (IQR) weekly darbepoetin-equivalent doses at baseline, Month 3, and Month 6 in each group. Change in weekly darbepoetin-equivalent dose from baseline to Month 6 was − 1.3 (− 10.0, 0.0) μg in the placebo group and 0.0 (− 7.5, 10.0) μg in the cholecalciferol group (*P* = 0.15).

**Table 2:** Hepcidin-25, haemoglobin, ESA resistance index, and inflammatory markers over the study period in the placebo and cholecalciferol groups.

	Group	Baseline	Day 3		Month 3		Month 6	
		Values	Values	*P*	Values	*P*	Values	*P*
Hepcidin-25 (ng/mL)		18.0 (7.5, 54.0)	13.4 (2.1, 24.9)	0.004	17.8 (9.1, 41.6)	0.5	19.7 (13.5, 42.0)	0.04
	VD	22.8 (5.2, 43.1)	15.1 (6.4, 31.2)		20.1 (11.1, 48.1)		22.0 (3.9, 41.4)	
IL-6 (pg/mL)		5.3 (2.5, 10.5)	4.6 (2.3, 11.4)	0.23	4.8 (3.3, 9.8)	0.29	4.5 (2.3, 7.7)	0.33
	VD	5.5 (2.8, 10.4)	3.9 (2.4, 6.8)		4.6 (2.9, 13.9)		4.2 (2.5, 8.2)	
TNF-α (pg/mL)		3.9 (3.0, 5.4)	4.5 (3.4, 5.6)	0.19	3.5 (2.8, 6.6)	0.13	3.9 (3.4, 4.5)	0.66
	VD	4.3 (3.3, 5.6)	4.5 (3.2, 5.5)		5.4 (3.0, 7.3)		3.8 (3.3, 4.7)	
ESA resistance index		0.03 (0.02, 0.05)	N/A		0.03 (0.03, 0.05)	0.73	0.03 (0.02, 0.04)	0.19
	VD	0.03 (0.02, 0.05)			0.03 (0.02, 0.05)		0.03 (0.02, 0.05)	
Haemoglobin (g/dL)		10.4 ± 1.0	N/A		10.4 ± 1.0	0.82	10.6 ± 1.1	0.23
	VD	10.8 ± 0.9			10.5 ± 1.0		10.4 ± 1.0	
Darbepoetin-equivalent ESA dose (μg)		20 (15, 30)	N/A		20 (15, 26)	0.94	19 (10, 23)	0.12
	VD	20 (11, 30)			20 (10, 30)		20 (11, 30)	
TSAT (%)		24 (16, 31)	N/A		25 (18, 33)	0.64	25 (20, 32)	0.34
	VD	22 (18, 29)			24 (17, 28)		23 (18, 30)	
Ferritin (ng/mL)		47 (21, 101)	N/A		57 (38, 86)	0.59	61 (31, 95)	0.16
	VD	62 (36, 95)			74 (31, 121)		42 (20, 89)	

Linear regression models including baseline values of the dependent variable were employed to examine whether the cholecalciferol group differed from the Placebo group. VD, cholecalciferol; ESA, erythropoiesis-stimulating agent; Weekly ESA dose of epoetin-β was converted to the equivalent darbepoetin-α dose (200 IU epoetin-β = 1 μg); ESA resistance index = Weekly ESA dose/ (Haemoglobin*Dry weight); IL-6, interleukin-6; TNF-α, tumour necrosis factor-α; TSAT, transferrin saturation.

Median (IQR) hepcidin levels at baseline were 21.6 (5.4, 43.6) ng/mL overall, but were largely different depending on the ESA type. Patients on thrice-weekly epoetin-α/β showed the highest hepcidin levels [median 43.1 (IQR 21.0, 60.6) ng/mL], followed by those on Wednesday/Thursday once-weekly darbepoetin [median 15.7 (IQR 9.1, 39.3) ng/mL] and Monday/Tuesday once-weekly darbepoetin [median 7.0 (IQR 0.6, 34.3) ng/mL]. No significant difference was observed between darbepoetin schedules.

Median (IQR) hepcidin levels of each group over the study period were shown in Fig. 2b and Table 2. After adjustment for baseline values, serum hepcidin levels at Day 3 were higher in the combined cholecalciferol than in the placebo group (*P* = 0.004), which was attenuated at Month 3 (*P* = 0.65). These findings were not significantly different between the thrice-weekly vs. once-monthly groups (*P* = 0.30 and 0.50, respectively), and were not significantly modified by age, gender, diabetes, ESA group (i.e., Monday/Tuesday darbepoetin, Wednesday/Thursday darbepoetin, and thrice-weekly epoetin-α/β), and baseline levels of 25(OH)D, hepcidin, ESA resistance index (Supplementary Fig. S1 online; *P*_interaction_ > 0.05 for all).

However, the cholecalciferol group showed lower serum hepcidin levels with greater ESA resistance index at Month 6 (Table 2). There were no significant between-group differences in haemoglobin, TNF-α, IL-6, iron saturation, or ferritin at any time point.

### Effects of cholecalciferol on serum 1,25(OH)_2_D and CKD-MBD parameters

**Figure 3:**
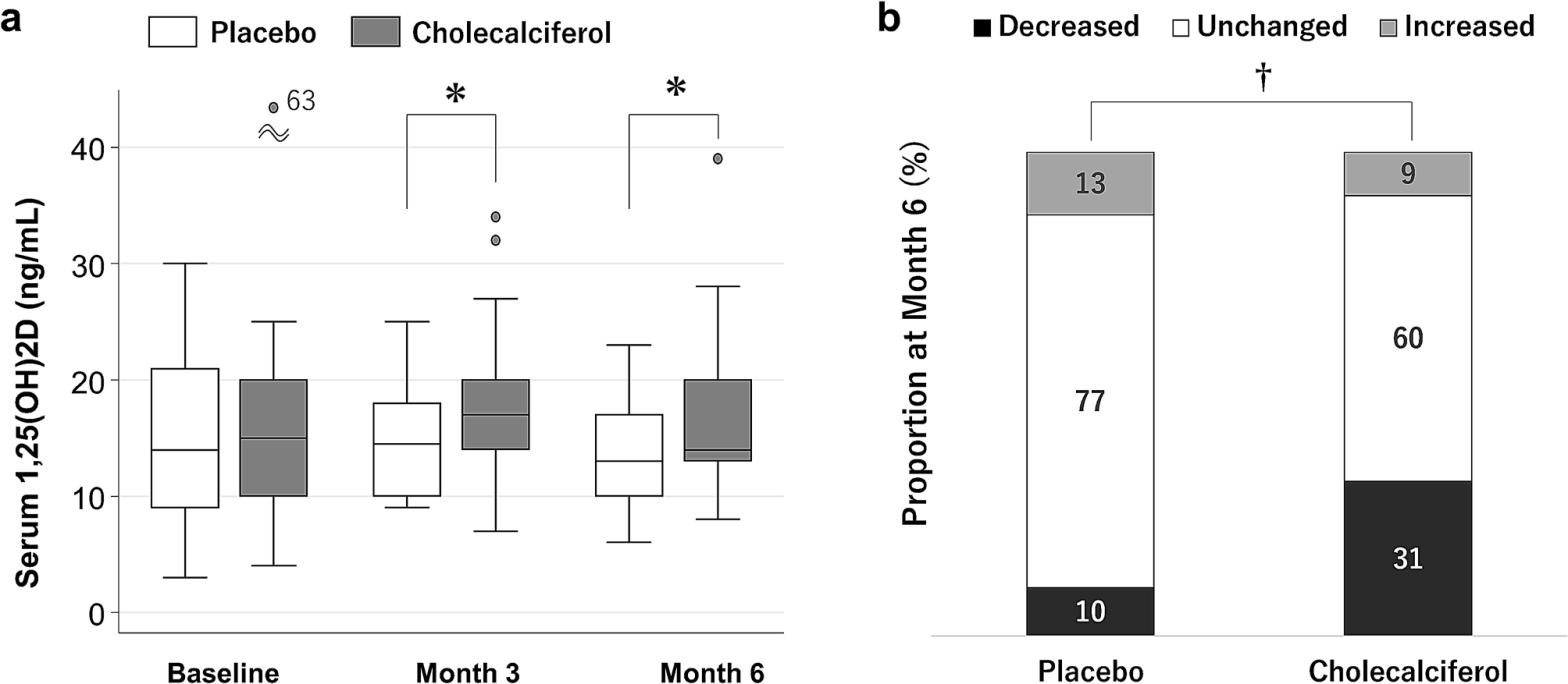
Changes in (**a**) serum 1,25(OH)_2_D levels and (**b**) doses of active vitamin D drugs. *P < 0.05 with adjustment for baseline values. ^**†**^*P* = 0.02 by the Wilcoxon–Mann–Whitney rank sum test.

**Figure 4:**
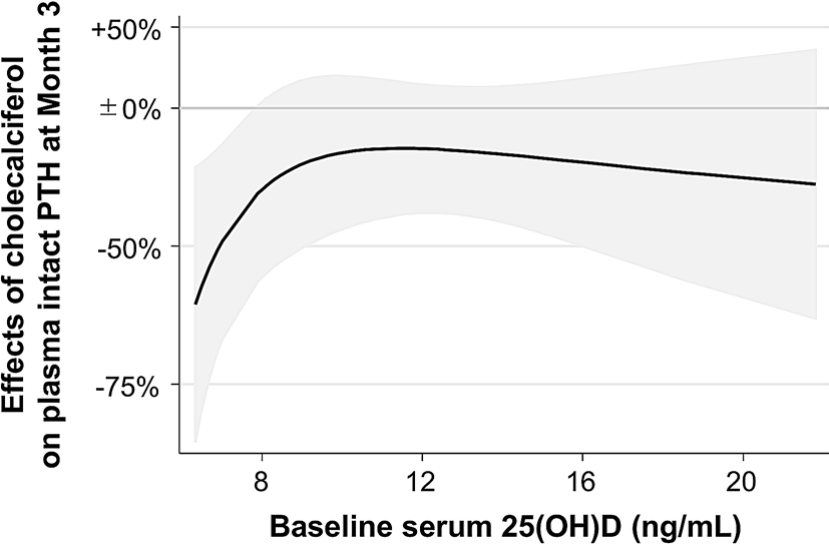
Effect of cholecalciferol on intact PTH levels at Month 3 according to baseline serum 25(OH)D levels. The multivariable fractional polynomials interaction analysis for baseline serum 25(OH)D levels was employed with adjustment for baseline intact PTH levels. Data were truncated at < 6 ng/mL and > 21 ng/mL.

Serum 1,25(OH)_2_D levels were higher in cholecalciferol group than the placebo group at Month 3 and 6 (Table 3 and Fig. 3a). The cholecalciferol group also more frequently reduced the dose of active vitamin D drugs from baseline to Month 6 (*P* = 0.02) (Fig. 3b). There were no significant between-group differences in serum levels of calcium, phosphorus, intact PTH, tartrate-resistant acid phosphatase 5b, or bone-specific ALP (Table 3). The multivariable fractional polynomials interaction analysis indicated that cholecalciferol supplementation decreased intact PTH at Month 3 to a greater extent with lower baseline serum 25(OH)D levels only if patients had baseline serum 25(OH)D levels < 8 ng/mL (Fig. 4).

**Table 3:** 1,25(OH)2D, calcium, phosphorus, and bone turnover markers over the study period in the placebo and cholecalciferol groups.

	Group	Baseline	Month 3		Month 6	
		Value	Value	P value	Value	P value
1,25(OH)_2_D (pg/mL)		14 (9, 21)	15 (10, 18)	0.02	13 (10, 17)	0.01
	VD	15 (10, 20)	17 (14, 20)		14 (13, 20)	
Calcium (mg/dL)		9.0 ± 0.6	8.9 ± 0.6	0.58	9.1 ± 0.8	0.09
	VD	9.0 ± 0.6	8.9 ± 0.6		8.9 ± 0.5	
Phosphate (mg/dL)		4.9 (4.1, 5.7)	5.1 (4.2, 5.7)	0.37	5.1 (4.1, 5.7)	0.79
	VD	5.0 (4.4, 5.7)	4.7 (4.3, 5.3)		4.7 (4.3, 5.6)	
Intact PTH (pg/mL)		93 (51, 183)	114 (77, 161)	0.39	94 (49, 186)	0.34
	VD	120 (65, 190)	113 (55, 180)		98 (66, 173)	
BSAP (μg/L)		25.9 (21.7, 33.7)	27.9 (21.3, 36.0)	0.59	22.2 (15.9, 28.1)	0.99
	VD	28.6 (23.2, 35.7)	30.4 (21.1, 37.0)		24.2 (17.7, 29.9)	
TRACP-5b (mU/dL)		386 (263, 610)	359 (284, 605)	0.45	371 (269, 556)	0.91
	VD	437 (294, 672)	365 (265, 655)		388 (248, 614)	

Linear regression models including baseline values of the dependent variable were employed to examine whether the cholecalciferol group differed from the Placebo group.

*VD* cholecalciferol, *BSAP* bone-specific alkaline phosphatase, *TRACP-5b* tartrate-resistant acid phosphatase 5b, *PTH* parathyroid hormone.

### Impact of vitamin D-related gene variants on serum 25(OH)D levels and the effect of cholecalciferol

Table 4 shows the prevalence and baseline serum 25(OH)D levels per SNPs on those selected vitamin D-related genes among 89 (93%) patients who provided informed consent to this genetic study. The T allele at VDR gene rs11568820 was associated with higher baseline serum 25(OH)D levels, while those alleles in the other candidate variants did not show significant association. However, none of the genetic variants significantly modified the effect of cholecalciferol on serum hepcidin level at Day 3, serum 25(OH)D and 1,25(OH)_2_D levels at Month 3, and ESA resistance index at Month 6 (*P*_trend_ for interaction > 0.05 for all).

**Table 4:** Prevalence of gene variants and baseline serum 25-hydroxyvitamin D levels in selected vitamin D-related genes.

	Prevalence	Median (IQR) ng/mL	*P* for trend
**CYP24A1 rs2209314**			
T/T	35%	11.4 (8.6, 13.9)	0.62
T/C	51%	10.9 (8.1, 14.3)	
C/C	15%	9.5 (6.0, 11.9)	
**DBP rs7041**			
A/A	58%	10.2 (7.6, 14.2)	0.53
A/C	37%	10.9 (8.6, 13.5)	
C/C	4%	12.3 (11.9, 13.2)	
**DBP rs2282679**			
T/T	54%	11.5 (9.6, 13.4)	0.93
T/G	36%	8.9 (7.6, 15.1)	
G/G	10%	10.8 (8.6, 14.5)	
**DBP rs12512631**			
T/T	56%	11.2 (8.5, 14.9)	0.86
T/C	37%	10.0 (6.9, 12.4)	
C/C	7%	11.9 (10.3, 12.1)	
**CYP2R1 rs2060793**			
G/G	42%	10.9 (8.5, 12.6)	0.92
A/G	48%	11.6 (8.3, 15.2)	
A/A	10%	9.2 (6.0, 11.4)	
**VDR rs11568820**			
C/C	44%	9.5 (7.2, 11.9)	0.02
T/C	43%	11.9 (8.2, 14.3)	
T/T	13%	14.3 (10.5, 15.8)	
**DHCR7 rs12785878**			
G/G	49%	11.0 (8.5, 15.0)	0.42
T/G	36%	10.6 (6.8, 12.7)	
T/T	15%	11.6 (8.8, 11.9)	
**TMPRSS6 rs855791**			
A/A	36%	11.3 (9.3, 13.7)	0.66
A/G	49%	10.9 (8.2, 13.7)	
G/G	15%	10.5 (6.4, 16.2)	

*CYP* cytochrome P450, *DBP* vitamin D binding protein, *VDR* vitamin D receptor, *DHCR7* 7-dehydrocholesterol reductase, *TMPRSS6* Transmembrane protease serine 6.

## Discussion

In this randomized controlled trial, cholecalciferol supplementation increased serum hepcidin levels at Day 3 among ESKD patients on MHD. Cholecalciferol supplementation also increased serum 1,25(OH)_2_D levels and decreased the dose of active vitamin D drugs whereas it did not change blood levels of calcium, phosphorus, and bone turnover markers. Both thrice-weekly and once-monthly regimen effectively increased serum 25(OH)D levels and showed equivalent effects. The T allele at VDR gene rs11568820 was associated with higher baseline serum 25(OH)D levels, but none of candidate variants in vitamin D-related genes modified the effects of cholecalciferol.

Our study result highlighted the importance of evidence-based medicine even for nutritional supplements, which may be particularly true with the ESRD population where a variety of metabolism are dysregulated. Indeed, our group have demonstrated that supplementation of vitamin B6 also worsened the response to ESA among MHD patients^27^. Nutritional supplementation in CKD and ESRD often lacks evidence to support, but is still prescribed under the assumption that it would be safe and/or inexpensive as in the general population. However, this assumption may not be true, and both potential risk and risk should be evaluated before it gains popularity with prevailing belief in clinical efficacy.

Several trials have evaluated the effects of nutritional vitamin D among MHD patients^28,29^. Reportedly, supplementation of cholecalciferol 100,000 IU/week increased mean 25(OH)D from 16 ng/mL to 43 mg/dL during 3 months^28^. Similarly, supplementation of ergocalciferol 50,000 IU/week increased mean 25(OH)D from 16 ng/mL to 41 ng/mL during 3 months in another trial^29^. In our study, mean 25(OH)D at baseline (11 ng/mL) were lower than those studies, which may reflect insufficient awareness of vitamin D deficiency among Japanese physicians because serum 25(OH)D measurement and cholecalciferol are reimbursed only for vitamin D deficiency-induced osteomalacia/rickets and denosumab-induced hypocalcaemia in Japan, respectively. Mean 25(OH)D among patients with cholecalciferol supplementation in the follow-up period was also lower than those studies, probably due to lower doses of nutritional vitamin D (weekly 9,000 IU cholecalciferol). Nevertheless, our study demonstrated that cholecalciferol supplementation significantly increased serum hepcidin levels at Day 3. More aggressive nutritional vitamin D supplementation among MHD patients, as done in those previous studies^28,29^, may result in a greater increase, but not a reduction, in serum hepcidin levels in the short term.

Increased serum hepcidin by cholecalciferol supplementation at Day 3 is contradicting the previous study of healthy subjects^16,30^, which may be attributed to ESA administration in patients with MHD. Exogenous erythropoietin has been shown to reduce serum hepcidin levels^31^. The underlying pathways in the reduction of serum hepcidin levels via ESA are not completely elucidated, but candidate mediators include CCAAT-enhancer-binding protein α (C/EBPα), a component of a family of transcription factors, and erythroferrone produced by erythroblasts in response to stimulated erythropoiesis. Reduced C/EBPα transcription was involved in suppression of hepcidin by erythropoietin^32^ whereas hepatocyte VDR activates C/EBPα^33^. Collectively, cholecalciferol may blunt the effect of ESA on hepcidin through increased C/EBPα. This finding appears particularly relevant to patients on epoetin-α/β. Although the effect of cholecalciferol on relative (or percent) change in serum hepcidin (evaluated as log-transformed levels) was not modified by the type and administration schedule of ESA, the absolute change was small for patients on darbepoetin because they have much lower baseline serum hepcidin levels than those on epoetin-α/β. This difference in serum hepcidin levels can be at least partly explained by the longer half-life of darbepoetin (~ 25 h) than epoetin (~ 8 h); darbepoetin leads to more sustained erythropoiesis compared to pulse-like stimulation by thrice-weekly epoetin α/β, and therefore maintains elevated serum erythroferrone levels up to 5 days (vs. 12 h with epoetin α/β)^34^. Further studies are needed to support these findings and identify the underlying mechanisms.

Although serum hepcidin levels turned to be lower in the cholecalciferol group than the placebo group at Month 6, it is less likely due to the direct effect of cholecalciferol for the following reasons. First, it may be simply a chance finding due to multiple comparisons with the secondary outcomes including serum hepcidin at Month 6. Second, parameters related to bone and mineral metabolism did not show any between-group differences at Month 6, suggesting that the effect of cholecalciferol was offset by the reduction of active vitamin D dose until then. Lastly, the change in ESA dose might have also affected serum hepcidin level. Although the between-group difference in ESA dose did not reach statistical significance in this small study (*P* = 0.12), this possibility cannot be ruled out given the potent effect of ESA on serum hepcidin.

Cholecalciferol did not affect calcium, phosphorus, or intact PTH levels as in previous RCTs of cholecalciferol^35,36^ or ergocalciferol^29,37^ among HD patients. It is notable, however, that cholecalciferol reduced active D dosage in our study. Furthermore, cholecalciferol suppressed intact PTH only among patients with very low 25(OH)D levels. Our patients had much lower baseline serum 25(OH)D levels than previous trials, which might have gave us an unprecedented chance to show such effects among vitamin D depleted patients. These results are in line with the findings in an open-label randomized clinical trial reported by Zheng et al.^38^. Therefore, nutritional vitamin D supplementation might be a cost-effective alternative to active vitamin D drugs especially among patients with vitamin D deficiency.

The minor allele (T) at VDR gene rs11568820 (Cdx) was associated with higher baseline serum 25(OH)D levels in our study. This finding is in contrast to healthy subjects where this genetic variant was not associated with serum 25(OH)D levels^39^. Vitamin D derived from the diet or cutaneous exposure to ultraviolet light is converted by 25-hydroxylase to 25(OH)D in the liver. In other words, serum 25(OH)D levels reflect intake and absorption of cholecalciferol, and outdoor activity. Given that the Cdx-T did not modify the effect of cholecalciferol supplementation on serum 25(OH)D levels, this genetic variant is less likely to be involved in intestinal absorption of vitamin D. However, further studies are needed to elucidate mechanisms underlying the association between gene polymorphism and 25(OH)D levels.

One of the strengths of this study is our study design, i.e., a placebo-controlled, double-blind RCT. Additionally, adherence to study drugs was ensured via direct observation by medical staffs at dialysis facilities. However, our study has also several limitations. First, the small sample size might have prevented us from detecting genetic variants that influence serum 25(OH)D levels or modify the effect of cholecalciferol. Second, the short study period did not allow us to evaluate long-term effects of supplementation of cholecalciferol beyond 6 months. Additionally, the primary endpoint, change in serum hepcidin levels, was not a hard clinical outcome.

## Conclusion

Supplementation of cholecalciferol does not decrease but rather slightly increases serum hepcidin levels in the short term among MHD patients. Both twice-weekly and once-monthly supplementation effectively increases serum 1,25(OH)_2_D levels and hence, reduces active vitamin D drugs without affecting calcium, phosphorus, and PTH levels. Further long-term, large studies are needed to examine the risk–benefit balance of cholecalciferol supplementation among MHD patients.

## Supplementary information

Supplementary information 1.

Supplementary information 2.

## Data Availability

The datasets of the current study are available from the corresponding author on reasonable request.

## Abbreviations

CKD: Chronic kidney disease
ESA: Erythropoiesis-stimulating agents
ERI: Erythropoietin resistance index
MHD: Maintenance haemodialysis
RCT: Randomized controlled trial
TRACP: Tartrate-resistant acid phosphatase
