# Supplementary material for: Effect of cholecalciferol on serum hepcidin and parameters of anaemia and CKD-MBD among haemodialysis patients: a randomized clinical trial
Source: Sci Rep. 2020 Sep 23;10:15500. doi: 10.1038/s41598-020-72385-w (PMC7512011; doi:10.1038/s41598-020-72385-w)

Supporting Information:

Effect of Cholecalciferol on Serum Hepcidin and Parameters of Anaemia and CKD-MBD among Haemodialysis Patients: A Randomized Clinical Trial

Author Names:

Yoshitsugu Obi, Satoshi Yamaguchi, Takayuki Hamano, Yusuke Sakaguchi, Akihiro Shimomura, Tomoko Namba-Hamano, Satoshi Mikami, Osamu Nishi, Motoko Tanaka, Akihito Kamoto, Yasue Obi, Naohisa Tomosugi, Yoshiharu Tsubakihara, and Yoshitaka Isaka.

Supplementary Table S1. Baseline characteristics of study participants across study groups

	Placebo (n=34)	Thrice-weekly cholecalciferol (n=32)	Once-monthly cholecalciferol (n=30)
Age (years)	67 (55, 73)	67 (60, 80)	67 (61, 78)
Male (%)	65%	66%	60%
Dialysis vintage (years)	5.8 (3.7, 12.1)	3.3 (2.4, 7.2)	4.8 (2.6, 8.0)
Post-dialysis weight (kg)	58.3 (51.0, 69.5)	61.2 (48.1, 69.3)	57.4 (50.5, 66.8)
Comorbidities			
Hypertension (%)	85%	78%	73%
Hyperlipidaemia (%)	44%	38%	33%
Diabetes (%)	47%	66%	37%
Cardiovascular disease (%)	35%	38%	33%
Liver disease (%)	6%	9%	3%
Laboratory measurements			
Haemoglobin (g/dL)	10.4 ± 1.0	10.6 ± 1.1	11.0 ± 0.6
Albumin (g/dL)	3.7 ± 0.3	3.7 ± 0.3	3.7 ± 0.3
Creatinine (mg/dL)	11.0 (8.7, 11.6)	10.4 (8.3, 12.4)	10.9 (9.5, 12.6)
Calcium (mg/dL)	9.0 ± 0.6	8.9 ± 0.6	9.0 ± 0.6
Phosphate (mg/dL)	4.9 (4.1, 5.7)	4.8 (4.2, 5.8)	5.2 (4.4, 5.7)
Intact PTH (ng/mL)	93 (51, 183)	123 (80, 179)	99 (53, 284)
BSAP (μg/L)	25.9 (21.7, 33.7)	30.7 (24.4, 37.6)	26.1 (20.2, 32.4)
TRACP-5b (mU/dL)	386 (263, 610)	442 (304, 609)	430 (258, 708)
25(OH)D (ng/mL)	12.1 (9.6, 15.1)	10.2 (6.7, 12.7)	9.7 (8.2, 12.1)
1,25(OH)$_2$D (pg/mL)	14 (9, 21)	15 (9, 19)	15 (12, 20)
TSAT (%)	24 (16, 31)	21 (17, 28)	26 (21, 35)
Ferritin (ng/mL)	47 (21, 101)	68 (36, 103)	63 (30, 114)
Medication			
Oral iron treatment (%)	3%	0%	7%
ESA type (%)			
Epoetin-α/β	21%	31%	33%
Darbepoetin-α	79%	69%	67%
ACE inhibitors or ARB (%)	53%	56%	50%
Calcium carbonate (%)	65%	25%	63%
NCC phosphate binders (%)	76%	78%	73%
Active vitamin D drugs (%)	85%	88%	87%
Cinacalcet (%)	26%	31%	30%

Data are presented as %, mean ± SD, or median (IQR) as appropriate.
Abbreviations: ACE, angiotensin converting enzyme; ARB, angiotensin II receptor blockers; BSAP, bone-specific alkaline phosphatase; ESA, erythropoiesis-stimulating agent; NCC, non-calcium containing; PTH, parathyroid hormone; TRACP-5b, tartrate-resistant acid phosphatase 5b.
ESA resistance index was calculated as weekly darbepoetin dose (μg)/ haemoglobin (g/dL)/ dry weight (kg) after converting epoetin-α/β dose to the equivalent darbepoetin-α dose (200 IU epoetin-α/β = 1μg darbepoetin-α).

<u>Supplementary Fig. S1.</u> Stratified analyses for the estimated effect of cholecalciferol on serum hepcidin-25 levels at Day 3 and Month 3

$P_{\text{interaction}}$ >0.05 for all subgroups.

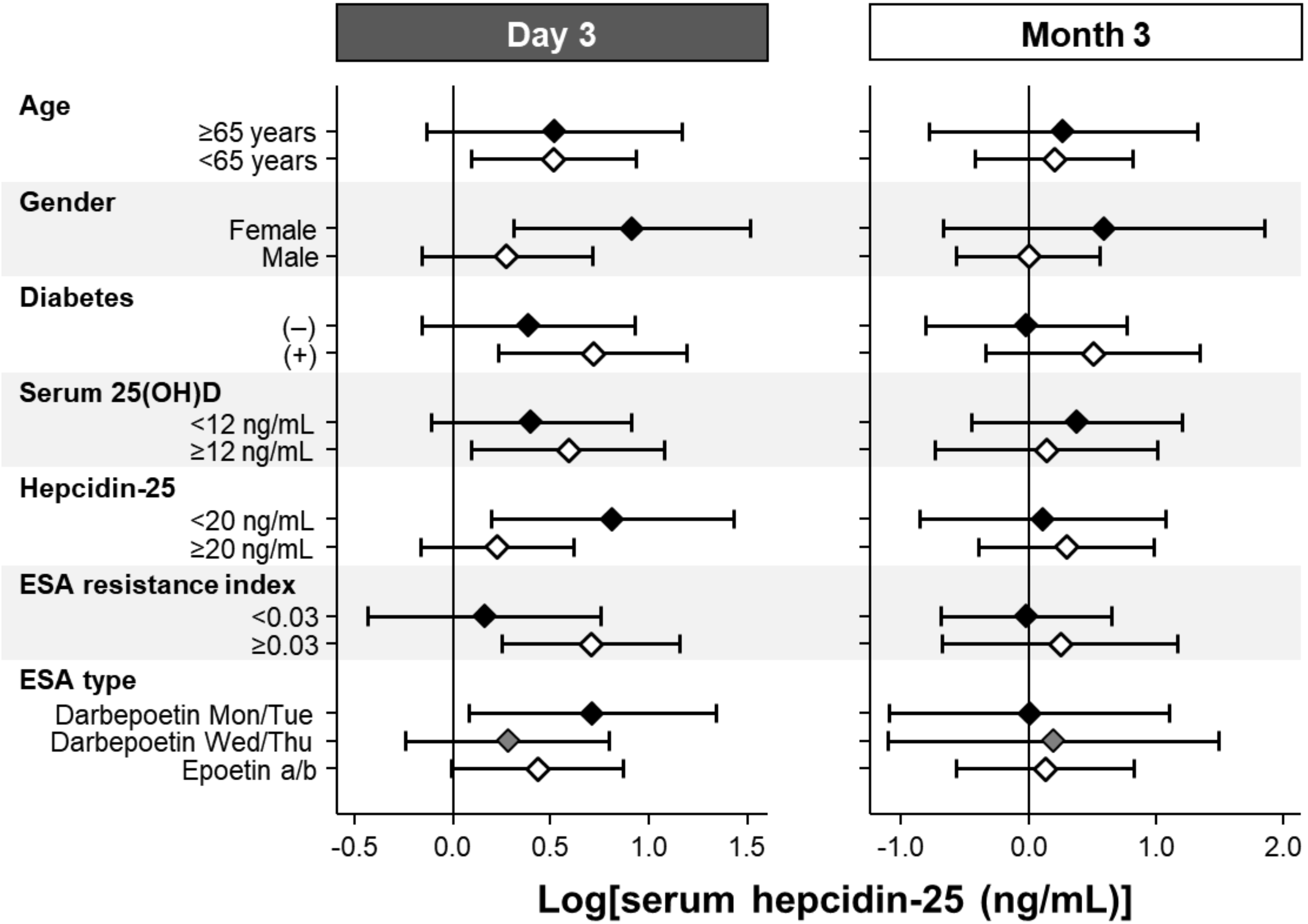

Supplement: Supplementary file 2 — Supplementary information 2. [file 41598_2020_72385_MOESM2_ESM.pdf]
